# Supplementary material for: Comparative Transcriptomics and Intestinal Microbiome Analysis Provide Insights into the Semi-Terrestrial Adaptation of Helice tientsinensis
Source: Animals (Basel). 2025 Apr 28;15(9):1244. doi: 10.3390/ani15091244 (PMC12070891; doi:10.3390/ani15091244)
Supplement: Supplementary file 1 [file animals-15-01244-s001.zip › Supplementary Materials/Table S1.docx]

**Table S1** BLAST analysis of non-redundant unigenes against public databases.

| Database | Number of annotated unigenes | | | Percentage of annoted unigenes（%） |
| --- | --- | --- | --- | --- |
| GO |  | 7,250 |  | 2.15 |
| KEGG |  | 9,330 |  | 2.77 |
| NR |  | 28,798 |  | 8.54 |
| PFAM |  | 8,448 |  | 2.51 |
| STRING |  | 1,203 |  | 0.36 |
| SWISSPROT |  | 7,299 |  | 2.16 |
