# Supplementary figures and images for: Comparative Transcriptomics and Intestinal Microbiome Analysis Provide Insights into the Semi-Terrestrial Adaptation of Helice tientsinensis
Source: Animals (Basel). 2025 Apr 28;15(9):1244. doi: 10.3390/ani15091244 (PMC12070891; doi:10.3390/ani15091244)

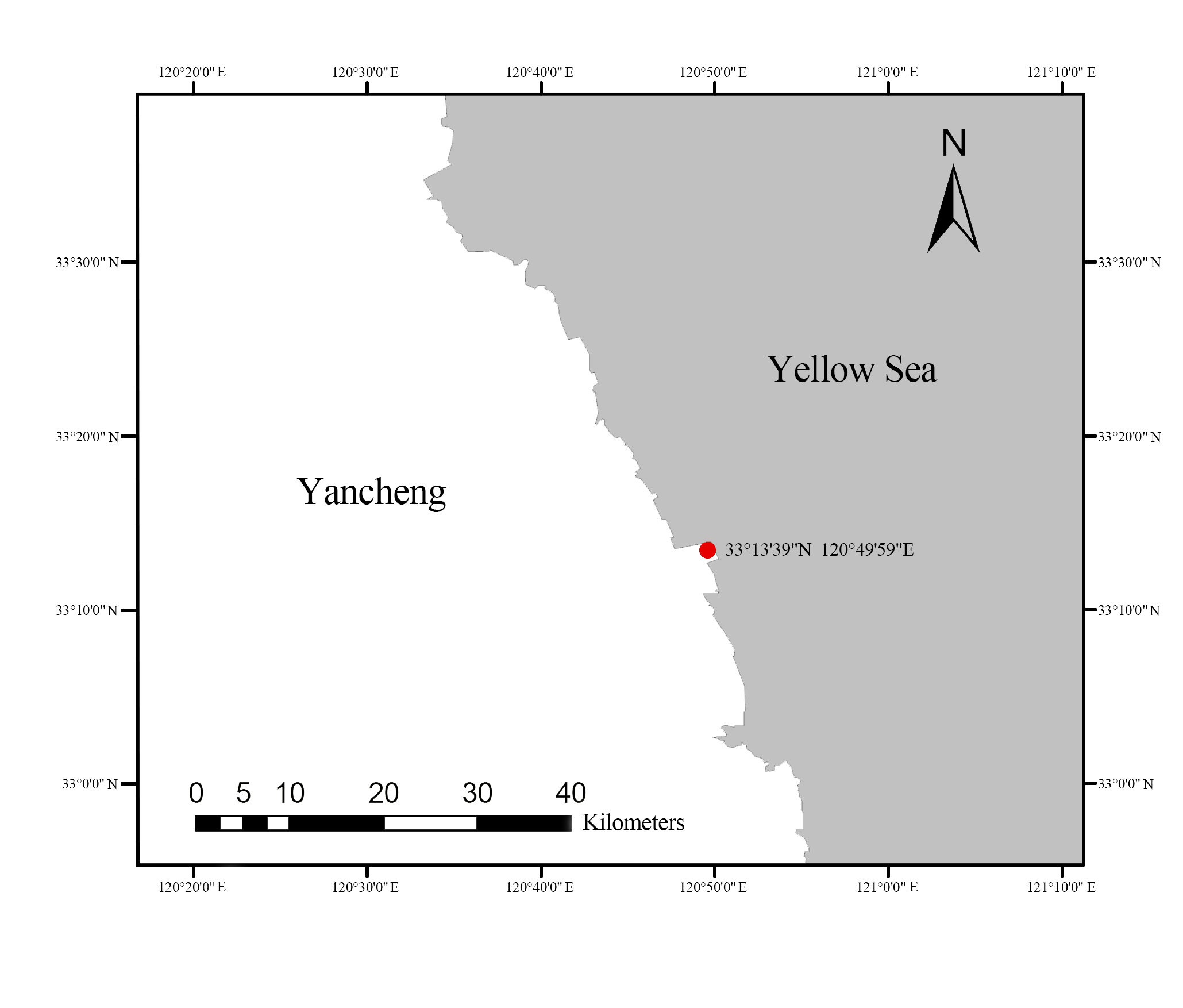

Supplement: Supplementary file 1 [file animals-15-01244-s001.zip › Supplementary Materials/Figure S1.png]

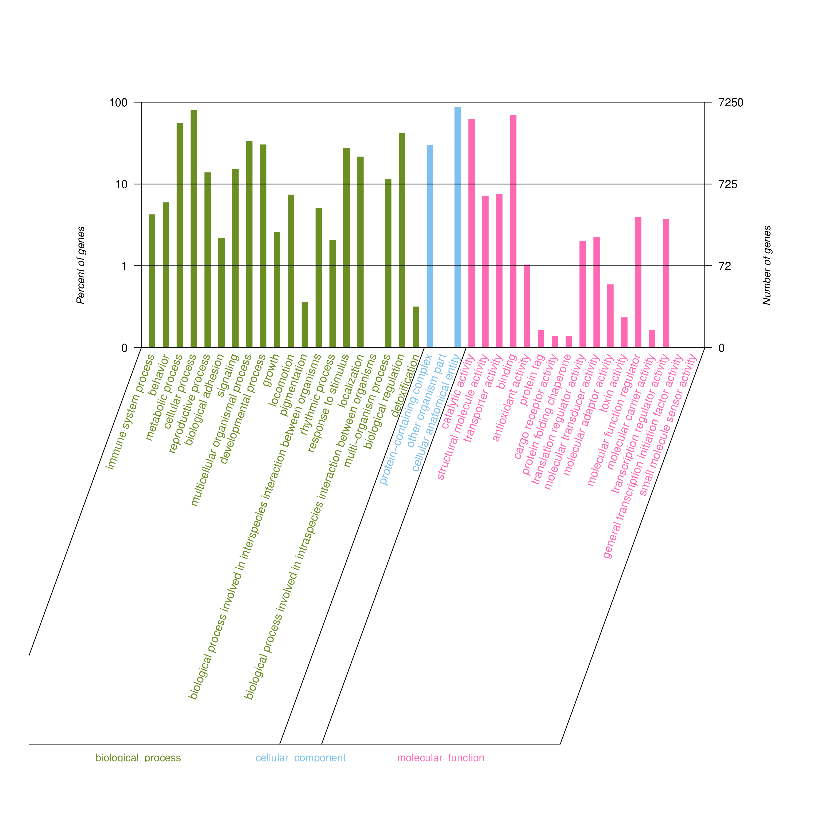

Supplement: Supplementary file 1 [file animals-15-01244-s001.zip › Supplementary Materials/Figure S2.tiff]

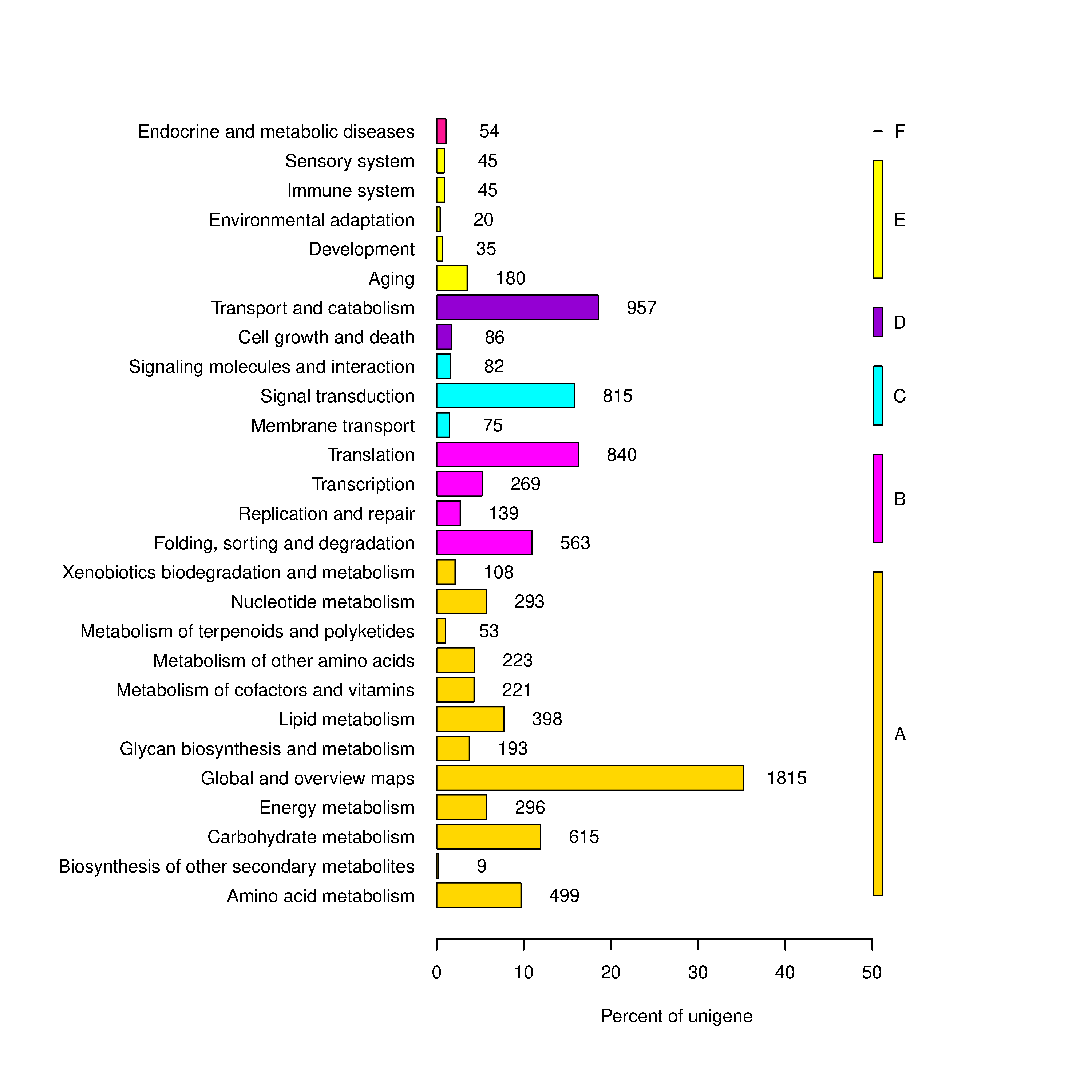

Supplement: Supplementary file 1 [file animals-15-01244-s001.zip › Supplementary Materials/Figure S3.tiff]

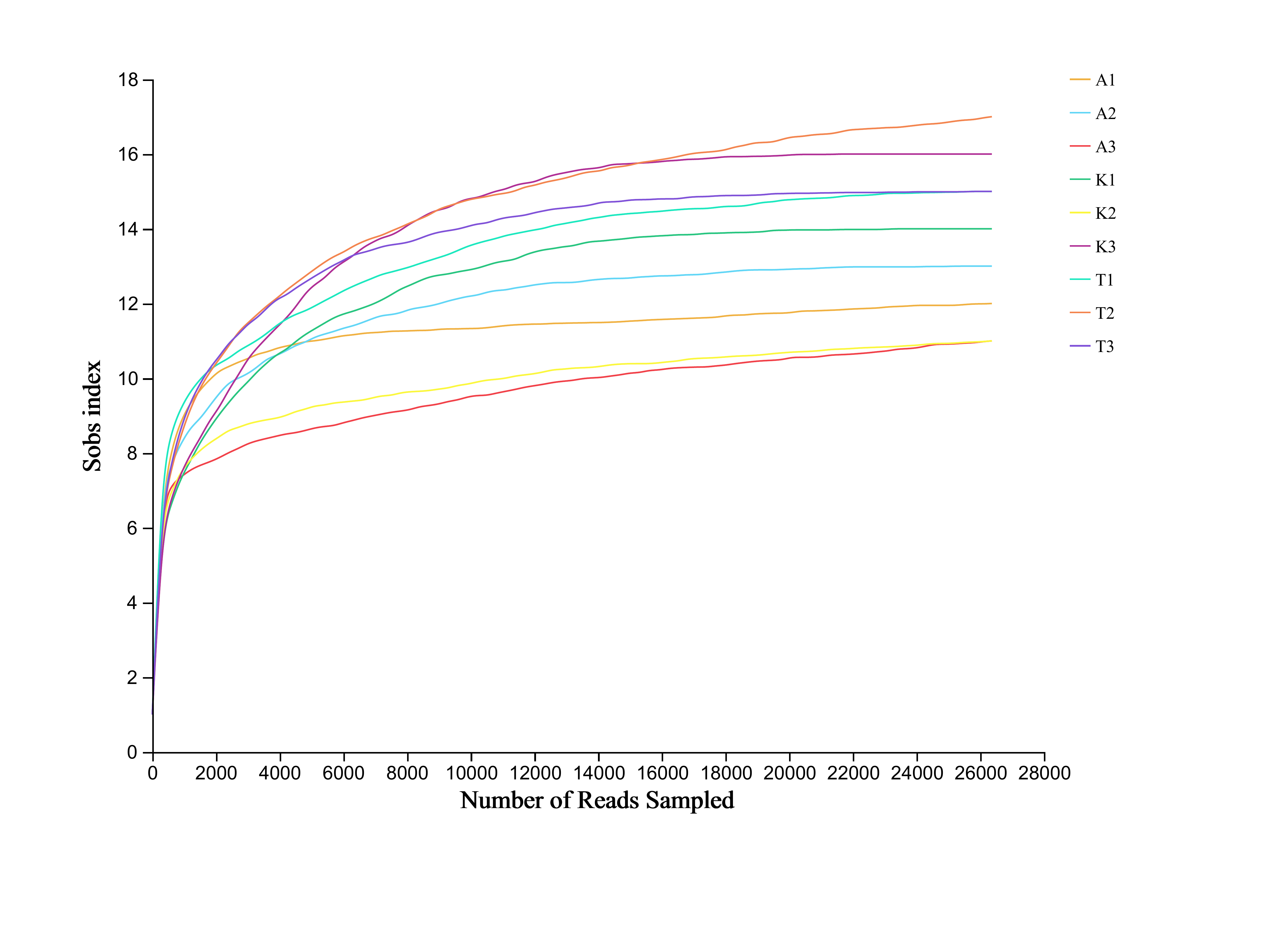

Supplement: Supplementary file 1 [file animals-15-01244-s001.zip › Supplementary Materials/Figure S4.tiff]
